# Causal Pathways from Enteropathogens to Environmental Enteropathy: Findings from the MAL-ED Birth Cohort Study

**DOI:** 10.1016/j.ebiom.2017.02.024

**Published:** 2017-03-08

**Authors:** Margaret N. Kosek, The MAL-ED Network Investigators, Tahmeed Ahmed, Zulfiquar Bhutta, Laura Caulfield, Richard Guerrant, Eric Houpt, Gagandeep Kang, Margaret Kosek, Gwenyth Lee, Aldo Lima, Benjamin J.J. McCormick, James Platts-Mills, Jessica Seidman, Laura Caulfield, Margaret Kosek, Gwenyth Lee, Benjamin J.J. McCormick, Jessica Seidman, Rebecca R. Blank, Michael Gottlieb, Stacey L. Knobler, Dennis R. Lang, Mark A. Miller, Karen H. Tountas, Zulfiqar A. Bhutta, Laura Caulfield, William Checkley, Richard L. Guerrant, Eric Houpt, Margaret N. Kosek, Dennis R. Lang, Carl J. Mason, Mark A. Miller, Laura E. Murray-Kolb, William A. Petri, Jessica C. Seidman, Tahmeed Ahmed, Pascal Bessong, Zulfiqar A. Bhutta, Rashidul Haque, Sushil John, Gagandeep Kang, Margaret N. Kosek, Aldo A.M. Lima, Estomih R. Mduma, Reinaldo B. Oriá, Prakash Sunder Shrestha, Sanjaya Kumar Shrestha, Erling Svensen, Anita K.M. Zaidi, Cláudia B. Abreu, Angel Mendez Acosta, Imran Ahmed, A.M. Shamsir Ahmed, Asad Ali, Ramya Ambikapathi, Leah Barrett, Aubrey Bauck, Eliwaza Bayyo, Ladaporn Bodhidatta, Anuradha Bose, J. Daniel Carreon, Ram Krishna Chandyo, Vivek Charu, Hilda Costa, Rebecca Dillingham, Alessandra Di Moura, Viyada Doan, Jose Quirino Filho, Jhanelle Graham, Christel Hoest, Iqbal Hossain, Munirul Islam, M. Steffi Jennifer, Shiny Kaki, Beena Koshy, Gwenyth Lee, Álvaro M. Leite, Noélia L. Lima, Bruna L.L. Maciel, Mustafa Mahfuz, Cloupas Mahopo, Angelina Maphula, Benjamin J.J. McCormick, Monica McGrath, Archana Mohale, Milena Moraes, Francisco S. Mota, Jayaprakash Muliyil, Regisiana Mvungi, Gaurvika Nayyar, Emanuel Nyathi, Maribel Paredes Olortegui, Reinaldo Oria, Angel Orbe Vasquez, William K. Pan, John Pascal, Crystal L. Patil, Laura Pendergast, Silvia Rengifo Pinedo, James Platts-Mills, Stephanie Psaki, Mohan Venkata Raghava, Karthikeyan Ramanujam, Muneera Rasheed, Zeba A. Rasmussen, Stephanie A. Richard, Anuradha Rose, Reeba Roshan, Barbara Schaefer, Rebecca Scharf, Jessica C. Seidman, Srujan L. Sharma, Binob Shrestha, Rita Shrestha, Suzanne Simons, Alberto M. Soares, Rosa M.S. Mota, Sajid Soofi, Tor Strand, Fahmida Tofail, Rahul J. Thomas, Ali Turab, Manjeswori Ulak, Vivian Wang, Ladislaus Yarrot, Pablo Peñataro Yori, Didar Alam, Ramya Ambikapathi, Caroline Amour, Cesar Banda Chavez, Sudhir Babji, Rosa Rios de Burga, Viyada Doan, Julian Torres Flores, Jean Gratz, Ajila T. George, Dinesh Hariraju, Alexandre Havt, Eric Houpt, Priyadarshani Karunakaran, Robin P. Lazarus, Ila F. Lima, Monica McGrath, Dinesh Mondal, Pedro H.Q.S. Medeiros, Rosemary Nshama, Josiane Quetz, Shahida Qureshi, Sophy Raju, Anup Ramachandran, Rakhi Ramadas, A. Catharine Ross, Mery Siguas Salas, Amidou Samie, Kerry Schulze, Jessica C. Seidman, E. Shanmuga Sundaram, Buliga Mujaga Swema, Dixner Rengifo Trigoso

**Affiliations:** a A.B. PRISMA, Iquitos, Peru; b Aga Khan University, Karachi, Pakistan; c Armed Forces Research Institute of Medical Sciences, Bangkok, Thailand; d Christian Medical College, Vellore, India; e Duke University, Durham, NC, USA; f Fogarty International Center/National Institutes of Health, Bethesda, MD, USA; g Foundation for the NIH, Bethesda, MD, USA; h Haydom Lutheran Hospital, Haydom, Tanzania; i icddr, b, Dhaka, Bangladesh; j Institute of Medicine, Tribhuvan University, Kathmandu, Nepal; k Johns Hopkins University, Baltimore, MD, USA; l The Pennsylvania State University, University Park, PA, USA; m Temple University, Philadelphia, PA, USA; n Universidade Federal do Ceara, Fortaleza, Brazil; o University of Bergen, Norway; p University of Illinois at Chicago, IL, USA; q University of Venda, Thohoyandou, South Africa; r University of Virginia, Charlottesville, VA, USA; s Walter Reed/AFRIMS Research Unit, Kathmandu, Nepal; t Haukeland University Hospital, Bergen, Norway

**Keywords:** Enteropathy, Undernutrition, Stunting, Enteropathogen, Child growth, Child health

## Abstract

**Background:**

Environmental enteropathy (EE), the adverse impact of frequent and numerous enteric infections on the gut resulting in a state of persistent immune activation and altered permeability, has been proposed as a key determinant of growth failure in children in low- and middle-income populations. A theory-driven systems model to critically evaluate pathways through which enteropathogens, gut permeability, and intestinal and systemic inflammation affect child growth was conducted within the framework of the Etiology, Risk Factors and Interactions of Enteric Infections and Malnutrition and the Consequences for Child Health and Development (MAL-ED) birth cohort study that included children from eight countries.

**Methods:**

Non-diarrheal stool samples (*N* = 22,846) from 1253 children from multiple sites were evaluated for a panel of 40 enteropathogens and fecal concentrations of myeloperoxidase, alpha-1-antitrypsin, and neopterin. Among these same children, urinary lactulose:mannitol (L:M) (*N* = 6363) and plasma alpha-1-acid glycoprotein (AGP) (*N* = 2797) were also measured. The temporal sampling design was used to create a directed acyclic graph of proposed mechanistic pathways between enteropathogen detection in non-diarrheal stools, biomarkers of intestinal permeability and inflammation, systemic inflammation and change in length- and weight- for age in children 0–2 years of age.

**Findings:**

Children in these populations had frequent enteric infections and high levels of both intestinal and systemic inflammation. Higher burdens of enteropathogens, especially those categorized as being enteroinvasive or causing mucosal disruption, were associated with elevated biomarker concentrations of gut and systemic inflammation and, via these associations, indirectly associated with both reduced linear and ponderal growth. Evidence for the association with reduced linear growth was stronger for systemic inflammation than for gut inflammation; the opposite was true of reduced ponderal growth. Although *Giardia* was associated with reduced growth, the association was not mediated by any of the biomarkers evaluated.

**Interpretation:**

The large quantity of empirical evidence contributing to this analysis supports the conceptual model of EE. The effects of EE on growth faltering in young children were small, but multiple mechanistic pathways underlying the attribution of growth failure to asymptomatic enteric infections had statistical support in the analysis. The strongest evidence for EE was the association between enteropathogens and linear growth mediated through systemic inflammation.

**Funding:**

Bill & Melinda Gates Foundation.

## Highlights

• Children living in these settings had a high prevalence of enteropathogens, high levels of intestinal inflammation, abnormal intestinal permeability, high markers of systemic inflammation, and postnatal acquired linear growth deficits when compared to children living in the US or Europe
• This study contributes empiric evidence to demonstrate that enteric infection alters both fecal markers of inflammation and permeability
• Current markers of enteropathy fail to account for a large portion of the observed shortfalls in linear growth in these populations, and markers of systemic inflammation appear as the most promising predictive biomarkers for identifying linear growth failure in children

Environmental enteropathy (EE) is hypothesized as a mediator of growth faltering, but few prospective studies have evaluated pathways linking enteropathogen exposure, intestinal inflammation and permeability, and growth. The MAL-ED study represents a novel analytical framework and explicitly evaluates multiple putative EE pathways in combination and using an unprecedented quantity of data. Despite evidence that gut inflammation and altered gut permeability are frequently present and that associations between enteropathogen exposure and gut dysfunction exist, the observed attributable effects of EE on growth faltering in young children were small.

## 1 Introduction

Traditionally, the evaluation of pathogenicity from enteric infections in the host has focused on the evaluation of defined diarrheal or acute gastrointestinal illnesses and the health outcomes associated with such illness perceived as binary, that is, either death or survival. It has been posited that the host response to frequent enteric infections alters the gut in a way that adversely affects the health status of the host even in the absence of diarrhea or acute gastrointestinal illness. The consequences of this altered host phenotype may have long term effects on child health and development potential. This condition is known as environmental enteropathy (EE). A proposed consequence of EE is reduced linear growth in children (Lunn, 2000, Keusch et al., 2013, Keusch et al., 2014), and EE may explain the less than expected effectiveness of nutritional interventions to improve growth in developing populations (Lunn et al., 1991, Humphrey, 2009, Korpe and Petri, 2012, Kosek et al., 2014). Several mechanisms have been proposed to explain how EE results in poorer nutritional status by reducing functional capacities of the gut. These include reduced absorptive capacity (Kelly et al., 2004, Menzies et al., 1999), increased permeability (Lunn et al., 1991), and chronic intestinal and systemic inflammation with resulting metabolic changes that affect nutrient and micronutrient availability and utilization (Campbell et al., 2003, Kosek et al., 2013).

Multiple physiological mechanisms by which enteropathogens can disrupt gut functioning have been identified (Guerrant et al., 1999, Berkes et al., 2003, Beltinger et al., 2008, Viswanathan et al., 2009, Kamada et al., 2013, Brown et al., 2015) although the long term consequences in settings where exposure to enteropathogens is intense and continuous (Platts-Mills et al., 2015) are poorly understood. Populations in low- and middle-income countries are also subject to other causes of growth failure, including inadequate dietary intake and frequent overt illness, any of which may influence both EE biomarkers and observed growth outcomes.

The collection of non-invasive biomarkers of EE is expanding, with different markers characterizing different aspects of gut physiology and integrity. The most widely used EE biomarker is the lactulose:mannitol (L:M) dual sugar test for intestinal permeability (Menzies et al., 1999, Denno et al., 2014), which has been used to demonstrate that altered gut permeability is related to the risk of stunting and is prevalent in environments with poor sanitation (Lunn et al., 1991, Lin et al., 2013, Weisz et al., 2012). Other EE bioassays available include fecal markers of gut inflammation (Campbell et al., 2004), intestinal growth factors (Peterson et al., 2013), and plasma markers of bacterial translocation (Naylor et al., 2015). Additionally, an increasing set of markers are becoming available that encompass systemic inflammation and amino acid and lipid metabolism (Campbell et al., 2003, Mondal et al., 2012, Hashimoto et al., 2012, Mayneris-Perxachs et al., 2016, Semba et al., 2016). Aligning the pathways indicted by this expanding collection of biomarkers with enteric infections and growth in early infancy and childhood across different populations is the subject of considerable current effort (Kosek et al., 2013, Peterson et al., 2013, Prendergast and Kelly, 2012).

**Fig. 1:**
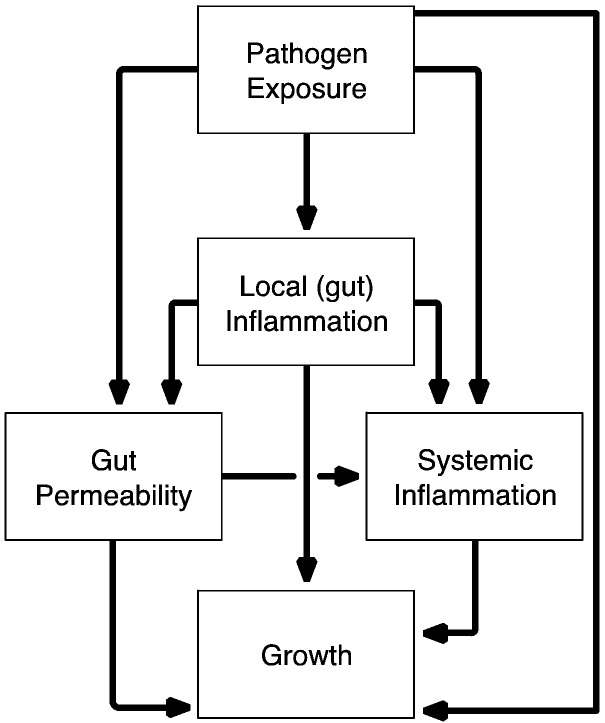
Conceptual model of the associations between pathogens, markers of gut function and inflammation, systemic inflammation and growth.

The Etiology, Risk Factors and Interactions of Enteric Infections and Malnutrition and the Consequences for Child Health and Development (MAL-ED) study was designed to assess the role of enteropathogens and other factors in growth faltering from birth to two years across eight sites (MAL-ED Network Investigators, 2014a). A central hypothesis of MAL-ED is that gut injury resulting in disruption of normal physiology is the key route by which enteropathogens contribute to malnutrition. Here, we use a causal systems model (a directed acyclic graph [DAG]) to test key theoretical pathways of the EE conceptual model and examine how enteropathogen infection results in impaired physical growth in infancy and early childhood (Fig. 1).

## 2 Methods

### 2.1 Study Design and Population

The MAL-ED study, conducted in eight diverse sites on three continents: Bangladesh (Dhaka: BGD), India (Vellore: INV), Nepal (Bhaktapur: NEB), and Pakistan (Naushero Feroze: PKN) in Southern Asia; Brazil (Fortaleza: BRF) and Peru (Loreto: PEL) in Latin America; and South Africa (Venda: SAV) and Tanzania (Haydom: TZH) in Sub-Saharan Africa. The study design is described in detail elsewhere (MAL-ED Network Investigators, 2014b). In brief, children were enrolled within 17 days of birth, but excluded if they had a birth weight < 1500 g, were very ill, or were non-singleton; or if their mother was < 16 years of age. Approximately 10 children were enrolled per month per site, with the goal of retaining ≥ 200 per site at 24 months of age (after loss to follow up). Data collection methods are described elsewhere for illness and treatment (Richard et al., 2014), infant feeding (Caulfield et al., 2014), and stool microbiology (Houpt et al., 2014). Each site obtained ethical approval from their respective institutions and written consent was obtained from participants.

Non-diarrheal stool samples collected monthly in the first year and quarterly in the second year were evaluated for > 40 pathogens using a standardized approach (Houpt et al., 2014). In addition to analyzing total number of pathogens detected per stool, we also categorized pathogens into five groups based on pathophysiology. Group I included viruses that cause limited mucosal disturbances (rotavirus, adenovirus and astrovirus). Group II included bacteria that are enteroinvasive or cause extensive mucosal disruption (*Campylobacter*, *Shigella*, *Salmonella*, *Plesiomonas*, *Yersinia*, enteroaggregative *E. coli* (EAEC), enteropathogenic *E. coli* (EPEC), enteroinvasive *E. coli* (EIEC) and *Aeromonas*). Group III was enterotoxigenic *E. coli* (ETEC), which is a cause of secretory diarrhea with only limited mucosal changes. *Cryptosporidium* (Group IV) and *Giardia* (Group V) were considered independently as organisms have both been shown to be associated with linear growth failure and prolonged and persistent carriage.

Three fecal biomarkers relating to aspects of gut inflammation and immunity (“local inflammation” in Fig. 1) were evaluated using the same non-diarrheal stool samples assayed for enteropathogens:(Kosek et al., 2014, Kosek et al., 2013) (1) myeloperoxidase (MPO, ng/mL) as a marker of neutrophil activity in the intestinal mucosa (Alpco, Salem, NH, USA); (Keusch et al., 2013) neopterin (NEO, nmol/L) to indicate T-helper cell 1 activity (GenWay Biotech, San Diego, CA, USA); and (Keusch et al., 2014) alpha-1-antitrypsin (AAT, mg/g) to indicate protein loss and intestinal permeability (Biovendor, Candler, NC, USA). Because diarrhea leads to stool dilution, fecal biomarker values were excluded if proximate to diarrheal symptoms (within seven days prior). Similarly, stools collected the day of or the day following the L:M test were excluded as this test is an osmotic laxative.

In addition to fecal biomarkers, urinary L:M testing (“gut permeability” in Fig. 1) was performed at three, six, nine, and 15 months, as described elsewhere (Kosek et al., 2014). Urine samples were processed using high-performance liquid chromatography and pulsed amperometric detection or ion chromatography (depending on study site). The results were converted into a sample-based *Z*-score (LMZ) to minimize age and sex trends. Data from the BRF cohort were used as the internal reference standard.

Finally, systemic inflammation was evaluated at seven, 15, and 24 months using alpha-1-acid glycoprotein (AGP) concentration in plasma. Incidence of acute lower respiratory infection (ALRI), diarrhea, fever (associated with neither ALRI nor diarrhea), and a composite for any of the three categorized illness episodes in the seven or 14 days preceding the blood collection were drawn from bi-weekly maternal reports. These were used to examine the influence of recent, non-diarrheal, overt illness on AGP concentration.

Monthly length (cm) and weight (kg) measures (Lohmann et al., 1988) were converted to *Z*-scores (LAZ, WAZ respectively) based on WHO 2006 standards (World Health Organization, 2006). The change (Δ) in LAZ and WAZ for each child (final minus initial value for each period) served as the outcome in all analyses, controlling for the initial value. Intense quality assurance review procedures identified bias within the PKN length measures; therefore, these data were excluded from the system analysis. PKN biomarker data were however, included in the evaluation of associations between pathogens and biomarkers.

### 2.2 Statistical Analysis

First, to maximally leverage the large size of the MAL-ED dataset and to place our results in the context of previous studies, we analyzed relationships between pathogens and fecal biomarker concentrations, between pathogens and LMZ scores, between LMZ scores and changes in anthropometry, and among potential sources of systemic inflammation not associated with gut enteropathy.

Linear mixed effects models were constructed to examine cross-sectional associations between individual pathogens and concentrations of each fecal biomarker. Specifically, the log concentrations of MPO, NEO, and AAT were modeled as functions of stool consistency (a categorical description of stool liquidity), linear and quadratic terms for child age (to capture age-related trends), the presence of individual pathogens (adjusting for the presence of other pathogens), and a random intercept for child nested in site (McCormick et al., 2016).

The same model structure was extended to evaluate associations between pathogen presence and LMZ scores, limiting the analyses to non-diarrheal stools collected at the same age as the L:M test. Additionally, changes in anthropometry (ΔLAZ and ΔWAZ) over three, six, and nine month windows starting at each L:M assay were evaluated as a function of the LMZ scores. Individual children nested within their respective site were treated as a random intercept to account for clustering at both the individual child and site levels.

To determine whether the concentration of AGP was related to overt illness in the seven or 14 days preceding blood collection, another linear mixed effects model was constructed with log-transformed AGP concentration as a function of age and illness (i.e., the presence of diarrhea, fever, and ALRI). A random intercept for child nested in site was included.

In addition to these linear regressions and given that disease systems composed of different interacting pathways lend themselves to causal graphical modeling (e.g., Fig. 1) (Pearl, 1995, Greenland et al., 1999) we constructed a DAG model to test hypothetical pathways between the presence of enteropathogens, biomarker concentrations, and changes in LAZ and WAZ. Combining all factors into a single system allowed for the explicit partition of associations into direct and indirect pathways.

**Fig. 2:**
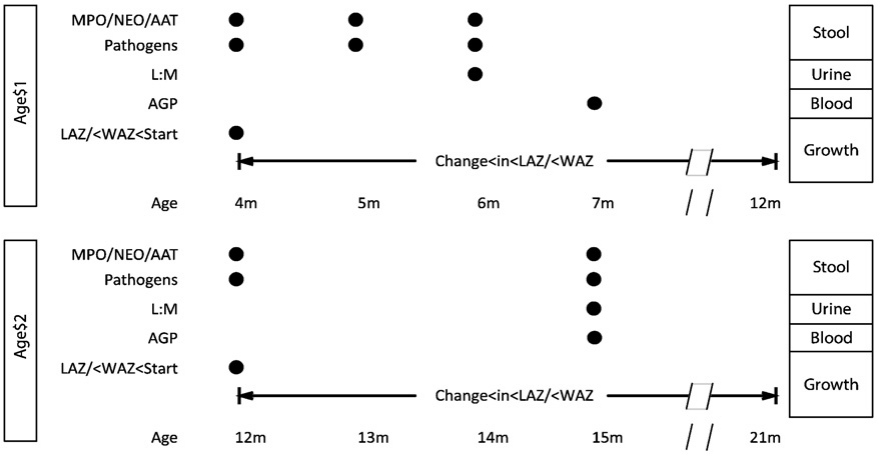
Timeline for collection of stool, urine, and blood samples and their respective biomarker assays that relate to changes in growth *Z*-scores.

Variables within this system were represented as conditionally independent, multivariate, generalized linear mixed models such that the probability of observing a given value for each variable was a function of other variables connected within the system (indicated by arrows in Fig. 1). To account for heterogeneity between sites, random effects for both site and child were added at every node. The DAG analysis focused on two time periods, 4 ≤ months ≤ 11 (Age 1) and 12 ≤ months ≤ 21 (Age 2), using the data collection schedule shown in Fig. 2 to capture temporal associations between events marked by the biomarkers. Specifically, pathogen data was coincident with collection of the fecal biomarkers (MPO, NEO, and AAT). Their collection preceded collection of L:M as alterations in gut permeability are a hypothesized result of inflammation. Measures of systemic inflammation then followed. The temporal window then extended beyond biomarker collection to assess associations with subsequent growth.

The net effects of both direct and indirect pathways were simulated from the fitted DAG. Sensitivities of ΔLAZ and ΔWAZ to changes in each biomarker were examined by fixing each biomarker to its observed mean concentration as well as one standard deviation higher or lower. The ΔLAZ and ΔWAZ were then simulated and the difference between their mean values when biomarkers were raised or lowered relative to when they were held at mean concentration were estimated.

The model was run in JAGS (version 3.4.0) to perform Markov Chain Monte Carlo simulations (Plummer, 2003). Further details are given in the Appendix.

### 2.3 Role of the Funding Source

The Bill & Melinda Gates Foundation did not play any role in the writing of the manuscript nor did the funders have of the study had any role in the study design, data collection, analysis, or interpretation of study results. The corresponding author had full access to all the data in the study and had final responsibility for the decision to submit for publication.

## 3 Results

Data from the entire cohort were included in the linear analyses for associations between pathogens and fecal biomarkers and LMZ, the changes in anthropometry following the L:M test, and between illness and AGP (Table 1, Table 2). Among the over 20,000 non-diarrheal stools tested for concentrations of MPO, NEO, and AAT, there was a trend for the concentration of each biomarker to decrease with increasing age. Within individual children; however, biomarker concentrations were highly variable across time, with intra-class correlations (ICC) of 0·07, 0·03, and 0·06 for MPO, NEO, and AAT respectively. Pearson correlation coefficients between the biomarkers were low (≤ 0·2) suggesting they measured different physiological insults.

**Fig. 3:**
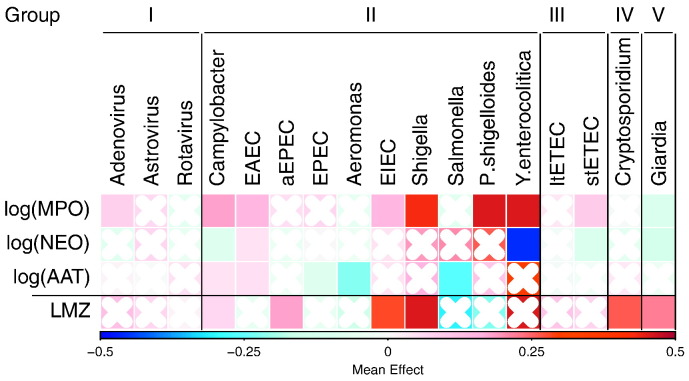
Effect of pathogens on the three fecal biomarkers (N non-diarrheal stools = 27,931) and the L:M test (N urine samples = 4476). The color represents the coefficient from a linear mixed effects model with pathogens found in the same stool as the fecal biomarkers or during the same month as the L:M test. Cells with crosses are not significant (p ≥ 0.05). Pathogens, within their groups (I˗V), are sorted by prevalence (high to low, left to right). In addition to the presence of individual pathogens, age was included using both linear and quadratic terms, stool consistency was included in the biomarker models, and child nested in site was included as a random intercept.

A comparison of associations between enteropathogens and the fecal biomarkers revealed that pathophysiological groups tended to show similar trends, with some prevalent pathogens being associated with either higher or lower biomarker concentrations (e.g., *Campylobacter* and EAEC were associated with higher concentrations of MPO and AAT, while *Giardia* was associated with lower concentrations of all three fecal biomarkers). The associations were more pronounced in some of the rarer pathogens (e.g., *Yersinia enterocolitica* was strongly associated with increased MPO and decreased NEO concentrations the few times it was detected) (Fig. 3). These exploratory models assumed additive effects of pathogens, though in many instances more than one pathogen was detected.

LMZ tended to be higher, indicating increased permeability, in children with pathogens detected, especially those with positive tests for *Cryptosporidium* (0·34 ± 0·08 for mean ± standard deviation, N = 134) and *Giardia* (0·20 ± 0·05, N = 457) (Fig. 3). However, there was no statistical support for LMZ relating to changes in either LAZ or WAZ (Table 3).

With respect to systemic inflammation, approximately half the measures of AGP were elevated (> 100 mg/dL), indicating common subclinical inflammation. Across the two time points when AGP was measured, high concentrations were dispersed between children rather than focused in an identifiable subset (ICC 0·16). Across 4257 AGP assays, neither symptoms of ALRI (which was uncommon at the time of the two blood draws) nor diarrhea were associated with concentrations of AGP (Table 4). Maternally-reported fever in the 14 days prior to the blood draw was associated with increased AGP concentration. Only the subset of MAL-ED children with complete data (i.e. with no missing observations across all variables) was included in the DAG (Table 1).

**Fig. 4:**
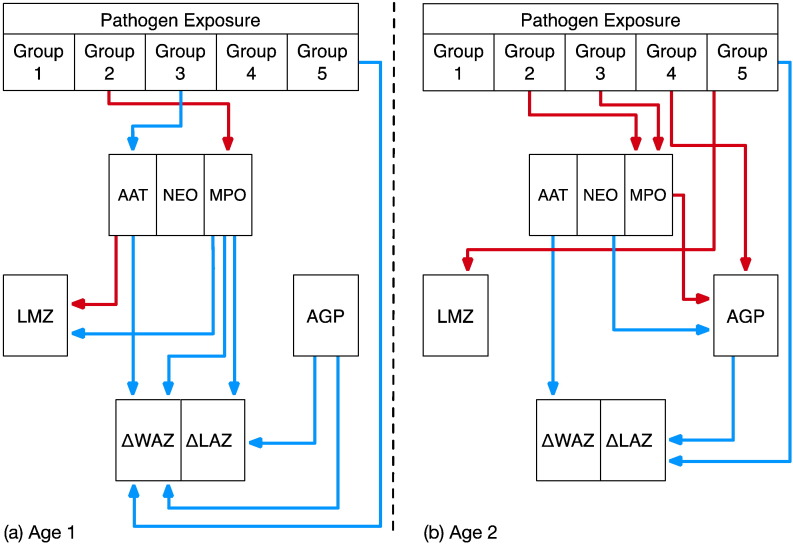
The model results for (a) Age 1 (4 ≤ months ≤ 11) and (b) Age 2 (12 ≤ months ≤ 21) using functional pathogen groupings and the specific pathways indicated by the individual fecal biomarkers as well as LMZ and AGP. Arrows show those relationships that had statistical support based on the 95% credibility interval. Red arrows indicate positive associations and blue arrows show negative associations. The pathogen groups reflect 1) viruses; 2) invasive bacteria; 3) non-invasive bacteria; 4) *Cryptosporidium*; and 5) *Giardia.*

Within the systems model, which included the pathogen groups, all biomarkers, and both growth outcomes, the invasive bacteria (Group II) showed positive associations with MPO for both ages (Fig. 4). Additionally, ETEC (Group III) infection was associated with lower concentrations of AAT at Age 1 and higher concentrations of MPO in older Age 2 children. At Age 1, AAT concentration was positively associated with LMZ, while MPO was negatively associated with LMZ (Fig. 4). In the older age period, MPO concentration was positively associated with AGP, while NEO was negatively associated with AGP.

Regarding associations between biomarkers and the two growth outcomes, ΔLAZ and ΔWAZ, the systems model revealed direct negative relationships between MPO and both ΔLAZ and ΔWAZ during Age 1 and negative associations between AAT and ΔWAZ in both age groups (Fig. 4) (i.e., the higher the concentration of MPO or AAT, the more restricted the growth over the respective period). There was no statistical support for an association between LMZ and either ΔLAZ or ΔWAZ in the system model (or for lactulose excretion, data not shown). Higher AGP concentrations were associated with decreased ΔLAZ at both ages and with ΔWAZ during Age 1 (Fig. 4). Because enteropathogens, particularly those associated with enteroinvasion or mucosal disruption (Group II), were directly related to increased MPO, they were thereby indirectly related to reduced growth. *Giardia* was associated directly with reduced growth, but not with the fecal biomarkers.

**Fig. 5:**
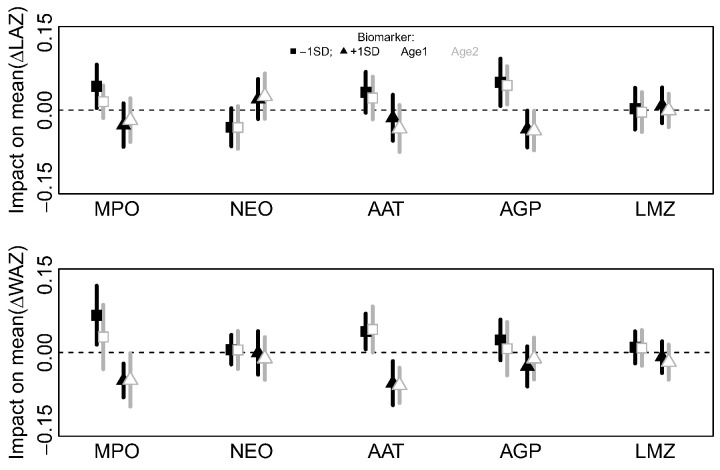
Sensitivity analysis of the DAG model (Fig. 4) to explore the effect of increasing (triangles) or decreasing (squares) the concentration of different biomarkers on mean ΔLAZ and ΔWAZ at the two age periods (Age 1, black; Age 2, gray). Symbols indicate the mean difference (lines, ± 1 standard deviation) in the mean simulated ΔLAZ and ΔWAZ when biomarkers are changed ± 1 standard deviation compared to a simulation using the mean observed biomarker value (i.e., the dotted horizontal line shows a difference of zero).

The effects of higher or lower biomarker concentrations relative to their observed means on ΔLAZ and ΔWAZ over each age period are shown in Fig. 5. None of the biomarkers had large mean effects on the average ΔLAZ or ΔWAZ. Of the effects on ΔLAZ, changing the log(AGP) concentration by ± 1SD produced the greatest difference (± 0·05 mean ΔLAZ), but little effect on the mean ΔWAZ (± 0·02 ΔWAZ) relative to the observed concentration of log(AGP). Changing the MPO concentration by ± 1SD had the second largest impact on ΔLAZ (+ 0·04 or − 0·03 for a decrease or increase in log(MPO) concentration respectively). The effect of increasing MPO concentration on ΔWAZ was marginally larger (− 0·05 ± 0·03), after which AAT had the second biggest impact on ΔWAZ (+ 0·04 when decreased and − 0·06 when log(AAT) was increased by 1SD).

## 4 Discussion

The systems model presented here is the first effort to explicitly combine enteropathogen exposure and different EE biomarkers (i.e., for gut permeability, gut inflammation, and systemic inflammation) into a single system and examine prospectively the pathways through which they relate to growth, thus providing an important proof of principal for a poorly defined condition. The temporal nature of the data allows for the exploration of causality and for the natural history of the biologic processes being measured to be interpreted with rigor. Our model is informed by a substantial quantity of structured longitudinal data on enteropathogens in non-diarrheal stools, illness history, intestinal inflammation and permeability, systemic inflammation, and growth across seven populations. The number of repeated measures over time and measures within the same individual is a particular strength of these data. Enteropathogen presence changes the concentration of contemporary biomarkers of gut immunity (NEO), inflammation (MPO), and permeability (AAT and LMZ). We find additional evidence that both enteropathogens and gut inflammation relate to systemic inflammation.

Our results yield several important findings. First, children living in these eight countries with differing epidemiologic settings have consistently and strikingly high concentrations of MPO (nearly 5–10 times the those seen in the USA) (Saiki, 1998) and nearly twice the concentration of AAT (Meyers et al., 1985). They also have notable elevations in AGP and intestinal permeability (L:M) that are two to three times higher than values in healthy populations (Kremer et al., 1988). Additionally, there was a high burden of enteropathogens detected even in the absence of overt diarrhea episodes (Platts-Mills et al., 2015). But most importantly, related MAL-ED analyses have demonstrated that, in the diverse epidemiologic settings of the study, growth velocities were low (MAL-ED Network Investigators, n.d.-a) leading to an increased prevalence of stunting by two years (MAL-ED Network Investigators, n.d.-b). The evidence we present here suggests that EE as measured by these markers contributes to, but does not appear to be the predominant driver of growth faltering.

As hypothesized, pathogen presence in non-diarrheal stool samples was associated with higher fecal MPO; this finding was also noted for AAT, although the AAT findings were evident only in younger children (aged 4˗11 months). When fecal biomarkers were evaluated for associations with individual pathogens, stronger signals of inflammation were noted in response to the rarely detected *Shigella* and *Yersinia* and the ubiquitous *Campylobacter*. The differential importance of pathogens in inducing EE is an area that requires additional attention, especially given the availability of newer technologies that allow for broad coverage and quantification of enteropathogens (Platts-Mills et al., 2015), and other strategies for determining which microbial taxa are inducing mucosal response in the gut (MAL-ED Network Investigators, n.d.-b). Such studies will enhance our understanding of the possible impact of etiology-specific interventions on EE.

Each of the three fecal biomarkers in this analysis have previously been associated with an elevated risk of stunting in the first year of life (George et al., 2015). Similar results have been found in other studies (Lin et al., 2013, Naylor et al., 2015). Here, we also find support for direct effects of gut inflammation (i.e., as measured by the three fecal biomarkers) on ΔLAZ or ΔWAZ. The evidence for an effect of systemic inflammation (i.e., as measured by AGP) is more compelling than that of the EE biomarkers. We found no evidence for an association between augmented intestinal permeability (i.e., as measured by LMZ) and future growth. Despite the popular use of L:M as a test of EE in cross sectional studies, its predictive power for future growth is less clear. While many studies have shown the L:M ratio to be associated with the contemporaneous LAZ (Lunn et al., 1991, Goto et al., 2009, Campbell et al., 2002), others have found (as we did) that it was not predictive of change in LAZ of children (Lin et al., 2013, Weisz et al., 2012). Given that AAT was associated with both gut permeability and subsequently to changes in growth (especially WAZ, both Age 1 and 2), it is possible that AAT more reliably reflects changes (or more severe changes) in permeability than the L:M test. The polar surface area of AAT greatly exceeds that of lactulose, and the size of the permeability defect may be important to delineate in greater detail using alternate probes. It is worth noting that other MAL-ED data (i.e., non-growth data) indicate the L:M test is predictive of impaired efficacy of oral polio vaccine (MAL-ED Network Investigators, n.d.-c). As such, the L:M test may capture other domains of EE not examined in these analyses.

Our results suggest that reductions in the exposure to pathogens, in particular to invasive bacteria that increase MPO, could reduce systemic inflammation (AGP). Recent evidence from another cohort study similarly highlighted the importance of systemic inflammation (Naylor et al., 2015). AGP interacts with bacterial lipopolysaccharide (LPS) (Moore et al., 1997), which is an indicator of bacterial translocation, and LPS bound to AGP is more rapidly cleared from the body than unbound LPS. Consequently, it was surprising to see that neither LMZ nor AAT were associated with AGP, as permeability leading to bacterial translocation has been a principal hypothesized component of EE (Lunn et al., 1991). Other markers of bacterial translocation are needed to confirm these findings.

Although our model indicates that specific enteropathogens alter gut inflammation and permeability, the effect sizes of the pathways from biomarkers to growth were smaller than anticipated (Humphrey, 2009, Brown et al., 2015). Based on available evidence, the contribution of EE to growth deficits as captured in these biomarkers is small relative to the accrued growth deficits in these populations (Lunn et al., 1991, Humphrey, 2009, MAL-ED Network Investigators, n.d, MAL-ED Network Investigators, n.d). The use of additional biomarkers or combinations of biomarkers and growth phenotypes (Naylor et al., 2015) or inclusion of metabolic markers may help to identify stronger support for links between the interactions of enteropathogen pressure and undernutrition on growth failure. Additionally, on-going work in this study population to understand links between the microbiome (Subramanian et al., 2014), host metabolism (Mayneris-Perxachs et al., 2016), and growth will expand our understanding of how microbial populations affect the nutritional status in these populations (Lunn, 2000, Brown et al., 2015, Kau et al., 2015, Blanton et al., 2016, Kosek et al., 2016).

The analytical framework described here is the first attempt to explicitly examine causal pathways of EE. Our results demonstrate that exposure to enteropathogens results in abnormal gut permeability, inflammation, systemic immune activation, and growth failure, but suggest that additional work incorporating other critical features of host metabolic status and the microbiome are needed to explain the gap between the insults attributable to EE and the observed cumulative acquired deficits of growth in children in these populations.

## Contributors

Margaret N. Kosek and Benjamin J.J. McCormick devised the model in discussion with Richard L. Guerrant, Laura E. Caulfield, Tahmeed Ahmed, Zulfiquar Bhutta, Gagandeep Kang, Aldo Lima, Eric Houpt, and James Platts-Mills. Benjamin J.J. McCormick ran the analyses. Margaret N. Kosek, Benjamin J.J. McCormick, Gwenyth Lee, and Jessica C. Seidman participated in the interpretation of the results and drafted the manuscript. The MAL-ED Investigators participated in the design, conduct, and analysis of the MAL-ED study and its results.

## Declaration of Interests

We declare that we have no conflicts of interest.

## Figures and Tables

**Table 1:** The number of samples collected and with complete data to yield observations in individual children included in the system model. The analysis of individual biomarkers includes all available observations.

		Age 1 (4 ≤ m ≤ 11)		Age 2 (12 ≤ m ≤ 21)	
		Collected	Complete	Collected	Complete
Samples	Blood	1503	1476	1536	1503
	Urine	1767	1601	1706	1535
	Non-diarrheal stools	4481	4285	2821	2676
	Anthropometry	15,272	15,257	16,232	16,215
Children	Total	2001		1873	
	Excluding PKN	1734		1617	
	Complete data	1059		1070	

**Table 2:** Observed characteristics of the subset of the MAL-ED population with complete data that were included in the system model. The mean and standard deviation (SD) of the continuous variables (anthropometry and the biomarkers) are shown along with the percentage of the discrete variables that were positive for at least one pathogen in each of the groups or the presence of maternally reported symptoms of ALRI and fever preceding the AGP assay and the percentage of stools that were coincident with different food intakes.

Continuous variables	Age 1		Age 2		Discrete variables		Age 1	Age 2
	Mean	± SD	Mean	± SD			% Positive	% Positive
LAZ start	− 0.97	1.13	− 1.05	1.05	Pathogens	Group 1	6.85	9.11
ΔLAZ	− 0.06	0.63	− 0.20	0.47		Group 2	56.76	65.86
WAZ start	− 0.53	1.15	− 0.61	1.21		Group 3	6.85	10.21
ΔWAZ	− 0.04	0.50	− 0.13	0.41		Group 4	3.43	5.10
log(MPO)	8.90	1.26	8.33	1.27		Group 5	3.36	18.35
log(NEO)	7.69	1.08	7.30	1.30	ALRI		1.71	1.94
log(AAT)	− 0.89	0.93	− 1.17	1.13	Fever		28.26	24.18
log(AGP)	4.61	0.40	4.63	0.36				
LMZ	0.35	0.87	0.40	1.12				

**Table 3:** The relationship between the LMZ and changes in LAZ and WAZ over a three, six, or nine month period from the age of the L:M test. L:M tests were performed at three, six, nine, and 15 months. Child nested in study site was considered a random intercept to account for repeated measures and site heterogeneity.

	Mean effect (± standard error)					
	ΔLAZ (months post L:M test)			ΔWAZ (months post L:M test)		
	3 m	6 m	9 m	3 m	6 m	9 m
Constant (3 m L:M)	− 0.21 (0.08)⁎, ⁎⁎	− 0.71 (0.20)⁎⁎⁎	− 1.08 (0.25)⁎⁎⁎	− 0.21 (0.08)⁎	− 0.71 (0.20)⁎⁎⁎	− 1.08 (0.25)⁎⁎⁎
Constant (6 m L:M)	− 0.12 (0.02)⁎⁎⁎	− 0.21 (0.02)⁎⁎⁎	− 0.19 (0.02)⁎⁎⁎	− 0.12 (0.02)⁎⁎⁎	− 0.21 (0.02)⁎⁎⁎	− 0.19 (0.02)⁎⁎⁎
Constant (9 m L:M)	− 0.24 (0.02)⁎⁎⁎	− 0.33 (0.02)⁎⁎⁎	− 0.27 (0.02)⁎⁎⁎	− 0.24 (0.02)⁎⁎⁎	− 0.33 (0.02)⁎⁎⁎	− 0.27 (0.02)⁎⁎⁎
Constant (15 m L:M)	− 0.25 (0.02)⁎⁎⁎	− 0.40 (0.02)⁎⁎⁎	− 0.33 (0.02)⁎⁎⁎	− 0.25 (0.02)⁎⁎⁎	− 0.40 (0.02)⁎⁎⁎	− 0.33 (0.02)⁎⁎⁎
LAZ/WAZ at start	− 0.24 (0.01)⁎⁎⁎	− 0.65 (0.01)⁎⁎⁎	− 0.82 (0.01)⁎⁎⁎	− 0.24 (0.01)⁎⁎⁎	− 0.65 (0.01)⁎⁎⁎	− 0.82 (0.01)⁎⁎⁎
L:M *Z*-score (3 m)	0.00 (0.02)	− 0.01 (0.01)	0.00 (0.01)	0.00 (0.02)	− 0.01 (0.01)	0.00 (0.01)
L:M *Z*-score (6 m)	− 0.03 (0.02)	0.03 (0.02)	− 0.01 (0.02)	− 0.03 (0.02)	0.03 (0.02)	− 0.01 (0.02)
L:M *Z*-score (9 m)	− 0.01 (0.02)	− 0.00 (0.02)	− 0.02 (0.02)	− 0.01 (0.02)	− 0.00 (0.02)	− 0.02 (0.02)
L:M *Z*-score (15 m)	− 0.02 (0.02)	0.00 (0.02)	0.00 (0.02)	− 0.02 (0.02)	0.00 (0.02)	0.00 (0.02)
N	5343	5213	5117	5343	5213	5117
N children	1650	1591	1563	1650	1591	1563
N sites	7	7	7	7	7	7
Variance (child)	0.03	0.37	0.61	0.03	0.37	0.61
Variance (site)	0.05	0.27	0.44	0.05	0.27	0.44
Variance (Residual)	0.29	0.18	0.13	0.29	0.18	0.13

⁎⁎⁎ p < 0·001.

⁎⁎ p < 0·01.

⁎ p < 0·05.

**Table 4:** The impact of recent overt symptoms on the log concentration of AGP based on samples at seven, 15, and 24 months. A linear mixed model of log(AGP) as a function of the presence or absence of overt symptoms in the 14 days preceding the blood draw. Child nested in site was treated as a random intercept.

Variable	Mean (± standard error)
Constant	4.58 (0.07)⁎⁎⁎, ⁎⁎, ⁎
Diarrhea	0.04 (0.05)
Fever	0.21 (0.04)⁎⁎⁎
ALRI	− 0.14 (0.07)
Age (months)	− 0.00 (0.00)
Diarrhea ∗ age	0.00 (0.00)
Fever ∗ age	0.00 (0.00)
ALRI ∗ age	0.02 (0.00)⁎⁎⁎

N	4257
N children	1801
N sites	8
Variance (child)	0.01
Variance (site)	0.03
Variance (residual)	0.15

⁎⁎⁎ p < 0·001.

⁎⁎ p < 0·01.

⁎ p < 0·05.

## Appendix A Supplementary data

MAL-ED Network Members. Image 1
