# Supplementary material for: Causal Pathways from Enteropathogens to Environmental Enteropathy: Findings from the MAL-ED Birth Cohort Study
Source: eBioMedicine. 2017 Mar 8;18:109–17. doi: 10.1016/j.ebiom.2017.02.024 (PMC5405169; doi:10.1016/j.ebiom.2017.02.024)
Supplement: Supplementary file 1 — MAL-ED Network Members. [file mmc1.docx]

**Appendix**

Causal pathways from enteropathogens to environmental enteropathy: findings from the MAL-ED birth cohort study

The MAL-ED Network Investigators^*^

*See appendix for list of authors.

Corresponding author:

Margaret Kosek, MD

Department of International Health

Johns Hopkins Bloomberg School of Public Health

615 N. Wolfe St E5608

Baltimore, MD 21205 USA

Tel: (410) 955-5932

E-mail: [mkosek@jhu.edu](mailto:mkosek@jhu.edu)

**MAL-ED Network Investigators**

**Writing group:**

Tahmeed Ahmed, Zulfiquar Bhutta, Laura Caulfield, Richard Guerrant, Eric Houpt, Gagandeep Kang, Margaret Kosek, Gwenyth Lee, Aldo Lima, Benjamin J.J. McCormick, James Platts-Mills, Jessica Seidman

**Data analysis group:**

Laura Caulfield, Margaret Kosek, Gwenyth Lee, Benjamin J.J. McCormick, Jessica Seidman

**Project management:**

Rebecca R Blank^7^, [rebecca.blank@gmail.com](mailto:rebecca.blank@gmail.com)

Michael Gottlieb^7^, [mgottlieb@fnih.org](mailto:mgottlieb@fnih.org)

Stacey L Knobler^6^, [Stacey.Knobler@nih.gov](mailto:Stacey.Knobler@nih.gov)

Dennis R Lang^6, 7^, [Lang4@fnih.org](mailto:Lang4@fnih.org)

Mark A Miller^6^, [Mark.Miller3@nih.gov](mailto:Mark.Miller3@nih.gov)

Karen H Tountas^7^, [ktountas@fnih.org](mailto:ktountas@fnih.org)

**Project technical subcommittee leadership:**

Zulfiqar A Bhutta^2^, [zulfiqar.bhutta@aku.edu](mailto:zulfiqar.bhutta@aku.edu)

Laura Caulfield^11^, [lcaulfi1@jhu.edu](mailto:lcaulfi1@jhu.edu)

William Checkley^11, 6^, [wcheckl1@jhmi.edu](mailto:wcheckl1@jhmi.edu)

Richard L Guerrant^18^, [guerrant@virginia.edu](mailto:guerrant@virginia.edu)

Eric Houpt^18^, [erh6k@virginia.edu](mailto:erh6k@virginia.edu)

Margaret N Kosek^11^, [mkosek@jhmi.edu](mailto:mkosek@jhmi.edu)

Dennis R Lang^6, 7^, [Lang4@fnih.org](mailto:Lang4@fnih.org)

Carl J Mason^3^, [carlmason@icloud.com](mailto:carlmason@icloud.com)

Mark A Miller^6^, [Mark.Miller3@nih.gov](mailto:Mark.Miller3@nih.gov)

Laura E Murray-Kolb^12^, [lem118@psu.edu](mailto:lem118@psu.edu)

William A Petri, Jr. ^18^, [wap3g@virginia.edu](mailto:wap3g@virginia.edu)

Jessica C Seidman^6^, [Jessica.Seidman@nih.gov](mailto:Jessica.Seidman@nih.gov)

**Study site lead investigators:**

Tahmeed Ahmed^9^, [tahmeed@icddrb.org](mailto:tahmeed@icddrb.org)

Pascal Bessong^17^, [pascal.bessong@univen.ac.za](mailto:pascal.bessong@univen.ac.za)

Zulfiqar A Bhutta^2^, [zulfiqar.bhutta@aku.edu](mailto:zulfiqar.bhutta@aku.edu)

Rashidul Haque^9^, [rhaque@icddrb.org](mailto:rhaque@icddrb.org)

Sushil John^4^, [rikkisush@cmcvellore.ac.in](mailto:rikkisush@cmcvellore.ac.in)

Gagandeep Kang^4^, [gkang@cmcvellore.ac.in](mailto:gkang@cmcvellore.ac.in)

Margaret N Kosek^11^, [mkosek@jhmi.edu](mailto:mkosek@jhmi.edu)

Aldo AM Lima^14^, [alima@ufc.br](mailto:alima@ufc.br)

Estomih R Mduma^8^, [estomih.mduma@haydom.co.tz](mailto:estomih.mduma@haydom.co.tz)

Reinaldo B Oriá^14^, [rbo5u@hscmail.mcc.virginia.edu](mailto:rbo5u@hscmail.mcc.virginia.edu)

Prakash Sunder Shrestha^10^, [prakashsunder@hotmail.com](mailto:prakashsunder@hotmail.com)

Sanjaya Kumar Shrestha^19^, [ShresthaSK@afrims.org](mailto:ShresthaSK@afrims.org)

Erling Svensen^20, 8^, [Erling.Svensen@cih.uib.no](mailto:Erling.Svensen@cih.uib.no)

Anita KM Zaidi^2^, [anita.zaidi@aku.edu](mailto:anita.zaidi@aku.edu)

**Data and sample collection and management:**

Cláudia B Abreu^14^, [claudia_beghini2004@yahoo.com.br](mailto:claudia_beghini2004@yahoo.com.br)

Angel Mendez Acosta^1^, [amendez@prisma.org.pe](mailto:amendez@prisma.org.pe)

Imran Ahmed^2^, [imran.ahmed@aku.edu](mailto:imran.ahmed@aku.edu)

AM Shamsir Ahmed^9^, [a.ahmed@uq.net.au](file:///C:\Users\Dennis\Downloads\a.ahmed@uq.net.au)

Asad Ali^2^, [asad.ali@aku.edu](mailto:asad.ali@aku.edu)

Ramya Ambikapathi^6^, [rambikapathi@gmail.com](mailto:rambikapathi@gmail.com)

Leah Barrett^18^, [ljbarrett@mindspring.com](https://mail.nih.gov/owa/redir.aspx?SURL=ylNCiUwlIdiB8hZ2YD5o5PjURw5tc7rmQlR7x314W-eQKQJUbwLTCG0AYQBpAGwAdABvADoAbABqAGIAYQByAHIAZQB0AHQAQABtAGkAbgBkAHMAcAByAGkAbgBnAC4AYwBvAG0A&URL=mailto%3aljbarrett%40mindspring.com)

Aubrey Bauck^11^, [abauck1@jhu.edu](mailto:abauck1@jhu.edu)

Eliwaza Bayyo^8^, [elb.bayo@gmail.com](https://kalender.uib.no/owa/redir.aspx?SURL=ub_2qWHkBTDGrgIHTjqmsjQyS_vF_c9EfXjHVufZnQVUeflVyALTCG0AYQBpAGwAdABvADoAZQBsAGIALgBiAGEAeQBvAEAAZwBtAGEAaQBsAC4AYwBvAG0A&URL=mailto%3aelb.bayo%40gmail.com)

Ladaporn Bodhidatta^3^, [ladapornb@afrims.org](mailto:ladapornb@afrims.org)

Anuradha Bose^4^, [abose@cmcvellore.ac.in](mailto:abose@cmcvellore.ac.in)

J Daniel Carreon^6^, [carreonj@mail.nih.gov](mailto:carreonj@mail.nih.gov)

Ram Krishna Chandyo^10^, [ram.chandyo@uib.no](mailto:ram.chandyo@uib.no)

Vivek Charu^6^, [vcharu@jhsph.edu](mailto:vcharu@jhsph.edu)

Hilda Costa^14^, [hildacosta@hotmail.com](mailto:hildacosta@hotmail.com)

Rebecca Dillingham^18^, [rd8v@virginia.edu](https://mail.nih.gov/owa/redir.aspx?SURL=JtHDEfZTn_iErPWJ-iQHJx2-1fDUbCv5MkGVAUrfHviQKQJUbwLTCG0AYQBpAGwAdABvADoAcgBkADgAdgBAAHYAaQByAGcAaQBuAGkAYQAuAGUAZAB1AA..&URL=mailto%3ard8v%40virginia.edu)

Alessandra Di Moura^14^, [ferrer.alessandra@yahoo.com.br](file:///C:\Users\doanv\Documents\MAL-ED\E-mail%20List\ferrer.alessandra@yahoo.com.br)

Viyada Doan^6^, [Viyada.Doan@nih.gov](mailto:Viyada.Doan@nih.gov)

Jose Quirino Filho^14, 6^, [jqf_ce@yahoo.com.br](mailto:jqf_ce@yahoo.com.br)

Jhanelle Graham^6^, [jhalexia@gmail.com](file:///C:\Users\doanv\Documents\MAL-ED\E-mail%20List\jhalexia@gmail.com)

Christel Hoest^6^, [christel.host@nih.gov](mailto:christel.host@nih.gov)

Iqbal Hossain^9^, [ihossain@icddrb.org](mailto:ihossain@icddrb.org)

Munirul Islam^9^, [mislam@icddrb.org](https://mail.nih.gov/owa/redir.aspx?SURL=koV1njEhW_TBe7SX1sujsl8Hy3EfA_bf4SB9Ncicl133kTgFhwXTCG0AYQBpAGwAdABvADoAbQBpAHMAbABhAG0AQABpAGMAZABkAHIAYgAuAG8AcgBnAA..&URL=mailto%3amislam%40icddrb.org)

M Steffi Jennifer^4^, [stefjeni.11@gmail.com](file:///C:\Users\doanv\Documents\MAL-ED\E-mail%20List\stefjeni.11@gmail.com)

Shiny Kaki^4^, [shinykaki@gmail.com](file:///C:\Users\doanv\Documents\MAL-ED\E-mail%20List\shinykaki@gmail.com)

Beena Koshy^4^, [beenakoshy1@rediffmail.com](mailto:beenakoshy1@rediffmail.com)

Gwenyth Lee^11^, [gwenyth.lee@gmail.com](mailto:gwenyth.lee@gmail.com)

Álvaro M Leite^14^, [alvaromadeiro@yahoo.com.br](file:///C:\Users\doanv\Documents\MAL-ED\E-mail%20List\alvaromadeiro@yahoo.com.br)

Noélia L Lima^14^, [noelialima30@yahoo.com.br](file:///C:\Users\doanv\Documents\MAL-ED\E-mail%20List\noelialima30@yahoo.com.br)

Bruna LL Maciel^14^, [brunalimamaciel@gmail.com](file:///C:\Users\doanv\Documents\MAL-ED\E-mail%20List\brunalimamaciel@gmail.com)

Mustafa Mahfuz^9^, [mustafa@icddrb.org](mailto:mustafa@icddrb.org)

Cloupas Mahopo^17^, [mahopotc@gmail.com](mailto:mahopotc@gmail.com)

Angelina Maphula^17^, [angelina.maphula@univen.ac.za](mailto:angelina.maphula@univen.ac.za)

Benjamin JJ McCormick^6^, [ben.mccormick@gmail.com](mailto:ben.mccormick@gmail.com)

Monica McGrath^6^, [mcgrath.monica@gmail.com](mailto:mcgrath.monica@gmail.com)

Archana Mohale^6^, [mohalea@mail.nih.gov](mailto:mohalea@mail.nih.gov)

Milena Moraes^14^, [milenamaia@hotmail.com](mailto:milenamaia@hotmail.com)

Francisco S Mota^14^, [sulivan.mota@iprede.org.br](mailto:sulivan.mota@iprede.org.br)

Jayaprakash Muliyil^4^, [jpmuliyil@gmail.com](file:///C:\Users\doanv\Documents\MAL-ED\E-mail%20List\jpmuliyil@gmail.com)

Regisiana Mvungi^8^, [regisiana@yahoo.com](file:///C:\Users\doanv\Documents\MAL-ED\E-mail%20List\regisiana@yahoo.com)

Gaurvika Nayyar^6^, [gaurvika@gmail.com](file:///C:\Users\doanv\Documents\MAL-ED\E-mail%20List\gaurvika@gmail.com)

Emanuel Nyathi^17^, [Emanuel.Nyathi@univen.ac.za](mailto:Emanuel.Nyathi@univen.ac.za)

Maribel Paredes Olortegui^1^, [mparedeso@prisma.org.pe](mailto:mparedeso@prisma.org.pe)

Reinaldo Oria^14^, [rbo5u@hscmail.mcc.virginia.edu](mailto:rbo5u@hscmail.mcc.virginia.edu)

Angel Orbe Vasquez^1^, [angel_orbe@hotmail.com](mailto:angel_orbe@hotmail.com)

William K Pan^5, 6^, [william.pan@duke.edu](mailto:william.pan@duke.edu)

John Pascal^8^, [johnagustinopaschal@gmail.com](https://kalender.uib.no/owa/redir.aspx?SURL=1qXGbGQk4CjeysFkypEyZpchGc-JZDOguiT2jn_SOs9UeflVyALTCG0AYQBpAGwAdABvADoAagBvAGgAbgBhAGcAdQBzAHQAaQBuAG8AcABhAHMAYwBoAGEAbABAAGcAbQBhAGkAbAAuAGMAbwBtAA..&URL=mailto%3ajohnagustinopaschal%40gmail.com)

Crystal L Patil^16^, [cpatil@uic.edu](mailto:cpatil@uic.edu)

Laura Pendergast^13^, [laura.pendergast@temple.edu](mailto:laura.pendergast@temple.edu)

Silvia Rengifo Pinedo^1^, [siguase36@hotmail.com](file:///C:\Users\Dennis\Downloads\siguase36@hotmail.com)

James Platts-Mills^18^, [jp5t@hscmail.mcc.virginia.edu](mailto:jp5t@hscmail.mcc.virginia.edu)

Stephanie Psaki^6^, [spsaki@popcouncil.org](file:///C:\Users\doanv\Documents\MAL-ED\E-mail%20List\spsaki@popcouncil.org)

Mohan Venkata Raghava^4^, [venkat@cmcvellore.ac.in](mailto:venkat@cmcvellore.ac.in)

Karthikeyan Ramanujam^4^, [karthikeyan05@yahoo.co.in](mailto:karthikeyan05@yahoo.co.in)

Muneera Rasheed^2^, [muneera.rasheed@aku.edu](mailto:muneera.rasheed@aku.edu)

Zeba A Rasmussen^6^, [Zeba.Rasmussen@nih.gov](mailto:Zeba.Rasmussen@nih.gov)

Stephanie A Richard^6^, [Stephanie.Richard@nih.gov](mailto:Stephanie.Richard@nih.gov)

Anuradha Rose^4^, [anurose@cmcvellore.ac.in](file:///C:\Users\doanv\Documents\MAL-ED\E-mail%20List\anurose@cmcvellore.ac.in)

Reeba Roshan^4^, [reebageorge@hotmail.com](mailto:reebageorge@hotmail.com)

Barbara Schaefer^12, 6^, [bas19@psu.edu](mailto:bas19@psu.edu)

Rebecca Scharf^18^, [rebeccascharf@virginia.edu](mailto:rebeccascharf@virginia.edu)

Jessica C Seidman^6^, [Jessica.Seidman@nih.gov](mailto:Jessica.Seidman@nih.gov)

Srujan L Sharma^4^, [srujan.sharma@gmail.com](mailto:srujan.sharma@gmail.com)

Binob Shrestha^19^, [binobs@afrims.org](mailto:binobs@afrims.org)

Rita Shrestha^10^, [ritas_12@yahoo.com](mailto:ritas_12@yahoo.com)

Suzanne Simons^12^, [sxs126@psu.edu](mailto:sxs126@psu.edu)

Alberto M Soares ^14^, [soaresam@ufc.br](file:///C:\Users\doanv\Documents\MAL-ED\E-mail%20List\soaresam@ufc.br)

Rosa MS Mota ^14^, [rosa@dema.ufc.br](file:///C:\Users\doanv\Documents\MAL-ED\E-mail%20List\rosa@dema.ufc.br)

Sajid Soofi^2^, [sajid.soofi@aku.edu](mailto:sajid.soofi@aku.edu)

Tor Strand^19, 15^, [Tors@me.com](file:///C:\Users\doanv\Documents\MAL-ED\E-mail%20List\Tors@me.com)

Fahmida Tofail^9^, [ftofail@icddrb.org](mailto:ftofail@icddrb.org)

Rahul J Thomas^4^, [rj_thomas99@yahoo.com](file:///C:\Users\doanv\Documents\MAL-ED\E-mail%20List\rj_thomas99@yahoo.com)

Ali Turab^2^, [turab.ali@aku.edu](mailto:turab.ali@aku.edu)

Manjeswori Ulak^10^, [manjeswori@gmail.com](mailto:manjeswori@gmail.com)

Vivian Wang^6^, [aijun.wang@nih.gov](mailto:aijun.wang@nih.gov)

Ladislaus Yarrot^8^, [ladisblacy@yahoo.com](mailto:ladisblacy@yahoo.com)

Pablo Peñataro Yori^11^, [pyori@jhsph.edu](mailto:pyori@jhsph.edu)

**Sample processing and management:**

Didar Alam^2^, [didar.alam@aku.edu](mailto:didar.alam@aku.edu)

Ramya Ambikapathi^6^, [rambikapathi@gmail.com](mailto:rambikapathi@gmail.com)

Caroline Amour^8^, [lyneamour@gmail.com](mailto:lyneamour@gmail.com)

Cesar Banda Chavez^1^, [cebchavez@yahoo.com](mailto:cebchavez@yahoo.com)

Sudhir Babji^4^, [sudhirbabji@cmcvellore.ac.in](mailto:sudhirbabji@cmcvellore.ac.in)

Rosa Rios de Burga^1^, [rosaburga@gmail.com](file:///C:\Users\doanv\Documents\MAL-ED\E-mail%20List\rosaburga@gmail.com)

Viyada Doan^6^, [Viyada.Doan@nih.gov](mailto:Viyada.Doan@nih.gov)

Julian Torres Flores^1^, [jflores@prisma.org.pe](file:///C:\Users\doanv\Documents\MAL-ED\E-mail%20List\jflores@prisma.org.pe)

Jean Gratz^18^, [jean.gratz@gmail.com](mailto:jean.gratz@gmail.com)

Ajila T George^4^, [agilatgeorge@gmail.com](mailto:agilatgeorge@gmail.com)

Dinesh Hariraju^4^, [dinesh85@gmail.com](mailto:dinesh85@gmail.com)

Alexandre Havt^14^, [ahavtbinda@gmail.com](mailto:ahavtbinda@gmail.com)

Eric Houpt^18^, [erh6k@virginia.edu](mailto:erh6k@virginia.edu)

Priyadarshani Karunakaran^4^, [priyadarshinicmc15@gmail.com](mailto:priyadarshinicmc15@gmail.com)

Robin P Lazarus^4^, [robin.lazarus@gmail.com](mailto:robin.lazarus@gmail.com)

Ila F Lima^14^, [ilafarm@yahoo.com.br](mailto:ilafarm@yahoo.com.br)

Monica McGrath^6^, [mcgrath.monica@gmail.com](mailto:mcgrath.monica@gmail.com)

Dinesh Mondal^9^, [din63d@icddrb.org](mailto:din63d@icddrb.org)

Pedro HQS Medeiros^14^, [phquintela@hotmail.com](file:///C:\Users\doanv\Documents\MAL-ED\E-mail%20List\phquintela@hotmail.com)

Rosemary Nshama^8^, [nshamarosemary@yahoo.com](https://kalender.uib.no/owa/redir.aspx?SURL=44eKywibT_VxkVYbqwNq_iu9dnbRDY3HdobxvhwMxEdUeflVyALTCG0AYQBpAGwAdABvADoAbgBzAGgAYQBtAGEAcgBvAHMAZQBtAGEAcgB5AEAAeQBhAGgAbwBvAC4AYwBvAG0A&URL=mailto%3anshamarosemary%40yahoo.com)

Josiane Quetz^14^, [jquetz@gmail.com](mailto:jquetz@gmail.com)

Shahida Qureshi^2^, [shahida.qureshi@aku.edu](mailto:shahida.qureshi@aku.edu)

Sophy Raju^4^, [sophyraju@gmail.com](mailto:sophyraju@gmail.com)

Anup Ramachandran^4^, [anuprama@yahoo.co.uk](file:///C:\Users\doanv\Documents\MAL-ED\E-mail%20List\anuprama@yahoo.co.uk)

Rakhi Ramadas^4^, [rakhi.ram9@gmail.com](mailto:rakhi.ram9@gmail.com)

A Catharine Ross^12^, [Acr6@psu.edu](https://mail.nih.gov/owa/redir.aspx?SURL=T_xGdajVnTwLywZ9Elh5LdwZz8FeOA4CJRlxVLMvT8SwxvYObALTCG0AYQBpAGwAdABvADoAQQBjAHIANgBAAHAAcwB1AC4AZQBkAHUA&URL=mailto%3aAcr6%40psu.edu)

Mery Siguas Salas^1^, [msiguas@prisma.org.pe](file:///C:\Users\doanv\Documents\MAL-ED\E-mail%20List\msiguas@prisma.org.pe)

Amidou Samie^17^, [samieamidou@yahoo.com](mailto:samieamidou@yahoo.com)

Kerry Schulze^11^, [kschulz1@jhu.edu](mailto:kschulz1@jhu.edu)

Jessica C Seidman^6^, [Jessica.Seidman@nih.gov](mailto:Jessica.Seidman@nih.gov)

Shanmuga Sundaram E^4^, [shanmugame@cmcvellore.ac.in](mailto:shanmugame@cmcvellore.ac.in)

Buliga Mujaga Swema^8^, [buligamujaga@yahoo.co.uk](https://kalender.uib.no/owa/redir.aspx?SURL=cnWumhcJ3YsHsQLZv_XfcEknGOYFFLkGvO1QIRmMpkZUeflVyALTCG0AYQBpAGwAdABvADoAYgB1AGwAaQBnAGEAbQB1AGoAYQBnAGEAQAB5AGEAaABvAG8ALgBjAG8ALgB1AGsA&URL=mailto%3abuligamujaga%40yahoo.co.uk)

Dixner Rengifo Trigoso^1^, [drengifo@prisma.org.pe](file:///C:\Users\doanv\Documents\MAL-ED\E-mail%20List\drengifo@prisma.org.pe)

**Affiliations**

^1^A.B. PRISMA, Iquitos, Peru

^2^Aga Khan University, Karachi, Pakistan

^3^Armed Forces Research Institute of Medical Sciences, Bangkok, Thailand

^4^Christian Medical College, Vellore, India

^5^Duke University, Durham, NC, USA

^6^Fogarty International Center/National Institutes of Health, Bethesda, MD, USA

^7^Foundation for the NIH, Bethesda, MD, USA

^8^Haydom Lutheran Hospital, Haydom, Tanzania

^9^icddr, b, Dhaka, Bangladesh

^10^Institute of Medicine, Tribhuvan University, Kathmandu, Nepal

^11^Johns Hopkins University, Baltimore, MD, USA

^12^The Pennsylvania State University, University Park, PA, USA

^13^Temple University, Philadelphia, PA, USA

^14^Universidade Federal do Ceara, Fortaleza, Brazil

^15^University of Bergen, Norway

^16^University of Illinois at Chicago, IL, USA

^17^University of Venda, Thohoyandou, South Africa

^18^University of Virginia, Charlottesville, VA, USA

^19^Walter Reed/AFRIMS Research Unit, Kathmandu, Nepal

^20^Haukeland University Hospital, Bergen, Norway

# Data

## Biomarker collection

Faecal stool samples, collected monthly for months 1-12 and quarterly thereafter. See details in.^1,2^

- Myeloperoxidase (MPO, ng/mL) (Alpco, Salem, NH, USA) as a marker of neutrophil activity in the intestinal mucosa;
- Neopterin (NEO, nmol/L) to indicate T-helper cell 1 activity (GenWay Biotech, San Diego, CA, USA);
- alpha-1-antitrypsin (AAT, mg/g) to indicate protein loss and intestinal permeability (Biovendor, Candler, NC, USA).

Urine samples were collected at 3, 6, 9, and 15 months. These were assayed for concentrations of lactulose (L) and mannitol (M) using high-performance liquid chromatography and pulsed amperometric detection or ion chromatography (depending on site). See details in.^1^

The results of the L:M ratio were converted into a sample-based Z-score (LMZ) to minimize age and sex trends in the values. Data from the Brazil cohort were used as the internal reference.

Blood samples take at 7 and 15 months were assayed for alpha-1- acid glycoprotein (AGP) using radial immunodiffusion.

## Visual inspection of variables

The figure below shows the bivariate relationships between observed variables (aggregating both age periods). The numbers in the upper-right corner show the Pearson’s correlation coefficient corresponding to the plot (in the lower-left corner):


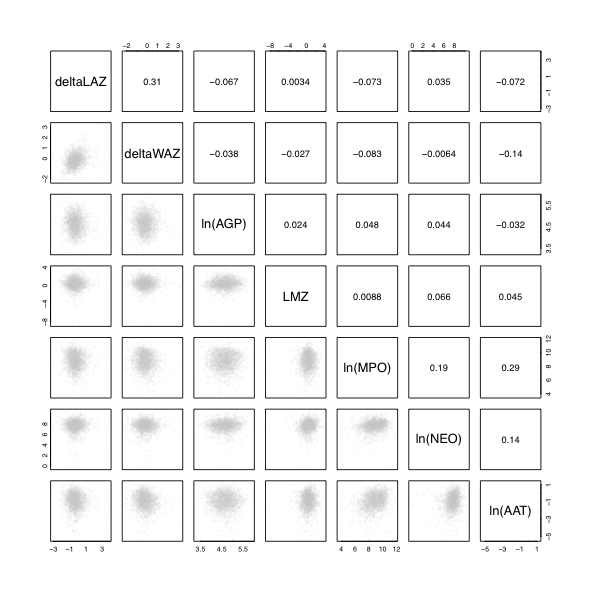


# Directed Acyclic Graph

A Directed Acyclic Graph (DAG) was constructed to model the relationships between variables within the EE system. The model was based on an expert description of putative relationships between enteropathogen exposure, biomarkers of gut functions, inflammation and growth outcomes.

The DAG was written in JAGS (version 3.4.0) using the code described below.

## Random Effects

A random variable (*rv.*[variable name]) was added for each variable in the model to account or site-level clustering, i.e. each site could have a different mean:

# M sites, i.e. 1 value per m^th^ site

# site random effects (intercept per variable)

rv.LAZ_change[M], #change in LAZ over time period

rv.LAZ_baseline[M], #LAZ at the start of the time period

rv.WAZ_change[M], #change in WAZ over time period

rv.WAZ_baseline[M], #WAZ at the start of the time period

rv.PathogenCount[M], #number of pathogens detected (NB. partitioned into 5 groups)

rv.log.AGP[M], #log(AGP)

rv.LMZ[M], #lactulose:mannitol Z-score (BRF internal reference)

rv.log.MPO[M], #log(MPO)

rv.log.NEO[M], #log(NEO)

rv.log.ALA[M], #log(ALA)

rv.ALRI[M], #presence of ALRI in 7d preceding AGP blood draw

rv.Fever[M], #presence of fever in 7d preceding AGP blood draw

Similarly, an additional random intercept (*rv2.*[variable name] in the code below) was added to each equation to account for child-level clustering. Given that children were sampled more than once (either multiple stools within an age period and potentially in both age periods), there was potential clustering at the child level in addition to that at a site level.

In both cases (site and child), the random effects were drawn from normal distributions with mean 0 and a precision parameter (1/standard deviation) using a hyperprior generated for each precision variable (*prec.rv.*[variable_name] or *rv2* for child-level hyperpriors) using a gamma distribution with shape 1 and rate 0.00005.

##site-wise random effects

prec.rv.LAZ_change ~dgamma(1,5E-05);

prec.rv.LAZ_baseline ~dgamma(1,5E-05);

prec.rv.WAZ_change ~dgamma(1,5E-05);

prec.rv.WAZ_baseline ~dgamma(1,5E-05);

prec.rv.StoolTests ~dgamma(1,5E-05);

prec.rv.PathogenGroup1 ~dgamma(1,5E-05);

prec.rv.PathogenGroup2 ~dgamma(1,5E-05);

prec.rv.PathogenGroup3 ~dgamma(1,5E-05);

prec.rv.PathogenGroup4 ~dgamma(1,5E-05);

prec.rv.PathogenGroup5 ~dgamma(1,5E-05);

prec.rv.log.AGP ~dgamma(1,5E-05);

prec.rv.LMZ ~dgamma(1,5E-05);

prec.rv.log.MPO ~dgamma(1,5E-05);

prec.rv.log.NEO ~dgamma(1,5E-05);

prec.rv.log.ALA ~dgamma(1,5E-05);

prec.rv.ALRI ~dgamma(1,5E-05);

prec.rv.Fever ~dgamma(1,5E-05);

The random effects for each site are thus given by (and child-level effects labelled *rv2*):

for(j in 1:M){

#Normal(mean=0, precision=hyperprior)

rv.LAZ_change[j] ~dnorm(0.0,prec.rv.LAZ_change);

rv.LAZ_baseline[j] ~dnorm(0.0,prec.rv.LAZ_baseline);

rv.WAZ_change[j] ~dnorm(0.0,prec.rv.WAZ_change);

rv.WAZ_baseline[j] ~dnorm(0.0,prec.rv.WAZ_baseline);

rv.StoolTests[j] ~dnorm(0.0,prec.rv.StoolTests);

rv.PathogenGroup1[j] ~dnorm(0.0,prec.rv.PathogenGroup1);

rv.PathogenGroup2[j] ~dnorm(0.0,prec.rv.PathogenGroup2);

rv.PathogenGroup3[j] ~dnorm(0.0,prec.rv.PathogenGroup3);

rv.PathogenGroup4[j] ~dnorm(0.0,prec.rv.PathogenGroup4);

rv.PathogenGroup5[j] ~dnorm(0.0,prec.rv.PathogenGroup5);

rv.log.AGP[j] ~dnorm(0.0,prec.rv.log.AGP);

rv.LMZ[j] ~dnorm(0.0,prec.rv.LMZ);

rv.log.MPO[j] ~dnorm(0.0,prec.rv.log.MPO);

rv.log.NEO[j] ~dnorm(0.0,prec.rv.log.NEO);

rv.log.ALA[j] ~dnorm(0.0,prec.rv.log.ALA);

v.ALRI[j] ~dnorm(0.0,prec.rv.ALRI);

rv.Fever[j] ~dnorm(0.0,prec.rv.Fever);

}#end site loop

## Fixed Effects

Bayesian inference works to estimate the probability of the data given the model. As such a combination of parameters that best match the observed data given the model are estimated presuming some probability process described by the model that generated the observed data. For example, a distribution of lengths might be assumed to come from a Gaussian process or the number of different pathogens detected in a stool sample may come from a Poisson process.

Here, the model loops through each observation, that is, the i^th^ observation of each variable, and attempts to find the combination of parameters that best describe the distributions from which a given observation was drawn.

Age was included as a variable to be modelled, and assumed to come from a Bernoulli distribution (equivalent to a binomial with 1 trial):

Age[i] ~ dbern(mu.Age[i]);

logit(mu.Age[i])<- Age.b0

The change in length was assumed to come from a normal distribution. Unlike age, which only had a constant intercept, the mean ΔLAZ was a function of the observed value for several other variables, given a child’s age (with a coefficient for the observed age group). Note that the random intercepts for the site (*rv*) and child (*rv2*) were included.

LAZ_change[i] ~ dnorm(mu.LAZ_change[i],prec.LAZ_change);

mu.LAZ_change[i]<-

(LAZ_change.b0 [Age[i]+1])

+ (LAZ_change.b1 [Age[i]+1])*LAZ_baseline[i]

+ (LAZ_change.b21[Age[i]+1])*PathogenGroup1[i]

+ (LAZ_change.b22[Age[i]+1])*PathogenGroup2[i]

+ (LAZ_change.b23[Age[i]+1])*PathogenGroup3[i]

+ (LAZ_change.b24[Age[i]+1])*PathogenGroup4[i]

+ (LAZ_change.b25[Age[i]+1])*PathogenGroup5[i]

+ (LAZ_change.b3 [Age[i]+1])*log.AGP[i]

+ (LAZ_change.b4 [Age[i]+1])*LMZ[i]

+ (LAZ_change.b5 [Age[i]+1])*log.MPO[i]

+ (LAZ_change.b6 [Age[i]+1])*log.NEO[i]

+ (LAZ_change.b7 [Age[i]+1])*log.ALA[i]

+ rv.LAZ_change[site[i]] + rv2.LAZ_change[pid[i]];

Similarly the LAZ at the start of the period is assumed to come from a normal distribution, the mean of which is a function of the baseline constant and random intercepts for site and child:

LAZ_baseline[i] ~ dnorm(mu.LAZ_baseline[i],prec.LAZ_baseline);

mu.LAZ_baseline[i]<-

(LAZ_baseline.b0[Age[i]+1])

+ rv.LAZ_baseline[site[i]] + rv2.LAZ_baseline[pid[i]];

WAZ was modelled in the same way as LAZ (including the same hypothesised relationships).

In contrast, the probability that a certain number of pathogens were detected is assumed to come from a Poisson process. The snippet of code below gives the function for pathogens in the virus group (group1) and this was repeated for the other pathogen groups or, if using the total number of pathogens detected, a single expression to describe the probability of observed the total pathogens in a given stool sample.

PathogenGroup1[i] ~ dpois(mu.PathogenGroup1[i]);

log(mu.PathogenGroup1[i])<-

(PathogenGroup1.b0[Age[i]+1])

+ rv.PathogenGroup1[site[i]] + rv2.PathogenGroup1[pid[i]];

The concentration of log(AGP) and the LMZ were assumed to be normally distributed:

log.AGP[i] ~ dnorm(mu.log.AGP[i],prec.log.AGP);

mu.log.AGP[i]<-

(log.AGP.b0 [Age[i]+1])

+ (log.AGP.b11[Age[i]+1])*PathogenGroup1[i]

+ (log.AGP.b12[Age[i]+1])*PathogenGroup2[i]

+ (log.AGP.b13[Age[i]+1])*PathogenGroup3[i]

+ (log.AGP.b14[Age[i]+1])*PathogenGroup4[i]

+ (log.AGP.b15[Age[i]+1])*PathogenGroup5[i]

+ (log.AGP.b2 [Age[i]+1])*log.MPO[i]

+ (log.AGP.b3 [Age[i]+1])*log.NEO[i]

+ (log.AGP.b4 [Age[i]+1])*log.ALA[i]

+ (log.AGP.b5 [Age[i]+1])*LMZ[i]

+ (log.AGP.b6 [Age[i]+1])*ALRI[i]

+ (log.AGP.b7 [Age[i]+1])*Fever[i]

+ rv.log.AGP[site[i]] + rv2.log.AGP[pid[i]];

LMZ[i] ~ dnorm(mu.LMZ[i],prec.LMZ);

mu.LMZ[i]<-

(LMZ.b0 [Age[i]+1])

+ (LMZ.b11[Age[i]+1])*PathogenGroup1[i]

+ (LMZ.b12[Age[i]+1])*PathogenGroup2[i]

+ (LMZ.b13[Age[i]+1])*PathogenGroup3[i]

+ (LMZ.b14[Age[i]+1])*PathogenGroup4[i]

+ (LMZ.b15[Age[i]+1])*PathogenGroup5[i]

+ (LMZ.b2 [Age[i]+1])*log.MPO[i]

+ (LMZ.b3 [Age[i]+1])*log.NEO[i]

+ (LMZ.b4 [Age[i]+1])*log.ALA[i]

+ rv.LMZ[site[i]] + rv2.LMZ[pid[i]];

The log-transformed concentration of each of the three faecal biomarkers were also assumed to come from normal distributions. For illustration, the code below shows the model for MPO, but that of AAT and NEO were modelled the same way:

log.MPO[i] ~ dnorm(mu.log.MPO[i],prec.log.MPO);

mu.log.MPO[i]<-

(log.MPO.b0 [Age[i]+1])

+ (log.MPO.b11[Age[i]+1]) *PathogenGroup1[i]

+ (log.MPO.b12[Age[i]+1]) *PathogenGroup2[i]

+ (log.MPO.b13[Age[i]+1]) *PathogenGroup3[i]

+ (log.MPO.b14[Age[i]+1]) *PathogenGroup4[i]

+ (log.MPO.b15[Age[i]+1]) *PathogenGroup5[i]

+ rv.log.MPO[site[i]] + rv2.log.MPO[pid[i]];

Both ALRI and fever were based on the presence of absence of symptoms preceding the blood draw. They were both modelled as Bernoulli distributions:

ALRI[i] ~ dbern(mu.ALRI[i]);

logit(mu.ALRI[i])<-

(ALRI.b0 + ALRI.b0a*Age[i])

+ rv.ALRI[site[i]] + rv2.ALRI[pid[i]];

## Priors

The precision of the normally distributed observed variables were, like the random effects, modelled with gamma-distributed hyper-priors, for example:

prec.log.AGP ~dgamma(1,5E-05);

All modelled coefficients were assumed to be normally distributed with mean 0 and a diffuse variance, for example

LAZ_change.b0[1] ~dnorm(0.0,0.001);

## Execution

The model was run in JAGS^3^ with a burn-in of 100,000 steps and the subsequently run for another 100,000 steps. This was judged adequate mixing based on visual inspection of 4 chains with different random initial seeds. Convergence was assessed with the Gelman-Rubin convergence diagnostic^4^.

## Parameter Estimates

The density plot of each parameter is given, where relevant showing both age periods. The 95% credibility interval is coloured in and a vertical line is included to indicate 0 (ie, assessing whether the 95% credibility interval includes 0).

The same numbers are presented in the tables below. Relationships highlighted in yellow did not include 0 in the 95% credibility interval.

**LAZ and WAZ nodes:**

| **RESPONSE** | **PREDICTOR** | **Age 1**  **95% credibility interval** | | | **Age 2**  **95% credibility interval** | | |
| --- | --- | --- | --- | --- | --- | --- | --- |
|  |  | **2.5%** | **50%** | **97.5%** | **2.5%** | **50%** | **97.5%** |
| delta LAZ | Intercept | -0.608 | 0.074 | 0.786 | -0.386 | 0.245 | 0.839 |
|  | start LAZ | -0.355 | -0.321 | -0.287 | -0.217 | -0.183 | -0.147 |
|  | Pathogen1 | -0.068 | 0.011 | 0.091 | -0.051 | 0.043 | 0.137 |
|  | Pathogen2 | -0.026 | -0.005 | 0.016 | -0.056 | -0.027 | 0.005 |
|  | Pathogen3 | -0.087 | -0.005 | 0.082 | 0.028 | 0.115 | 0.208 |
|  | Pathogen4 | -0.043 | 0.072 | 0.190 | -0.207 | -0.081 | 0.047 |
|  | Pathogen5 | -0.185 | -0.090 | 0.008 | -0.126 | -0.070 | -0.013 |
|  | AGP | -0.214 | -0.116 | -0.019 | -0.241 | -0.146 | -0.040 |
|  | LMZ | -0.043 | 0.000 | 0.043 | -0.029 | 0.004 | 0.038 |
|  | MPO | -0.084 | -0.046 | -0.007 | -0.038 | -0.005 | 0.030 |
|  | NEO | -0.013 | 0.034 | 0.080 | -0.045 | -0.010 | 0.024 |
|  | AAT | -0.068 | -0.016 | 0.038 | -0.078 | -0.037 | 0.003 |
| start LAZ | Intercept | -1.357 | -0.936 | -0.479 | -1.721 | -1.295 | -0.842 |
| delta WAZ | Intercept | 0.372 | 0.982 | 1.574 | -0.834 | -0.257 | 0.317 |
|  | start WAZ | -0.183 | -0.154 | -0.122 | -0.227 | -0.198 | -0.168 |
|  | Pathogen1 | -0.055 | 0.017 | 0.092 | -0.111 | -0.027 | 0.060 |
|  | Pathogen2 | -0.016 | 0.003 | 0.023 | -0.035 | -0.008 | 0.021 |
|  | Pathogen3 | -0.074 | 0.003 | 0.085 | -0.067 | 0.017 | 0.105 |
|  | Pathogen4 | -0.044 | 0.066 | 0.178 | -0.103 | 0.015 | 0.128 |
|  | Pathogen5 | -0.197 | -0.110 | -0.020 | -0.077 | -0.023 | 0.032 |
|  | AGP | -0.188 | -0.101 | -0.011 | -0.093 | 0.000 | 0.097 |
|  | LMZ | -0.054 | -0.016 | 0.024 | -0.039 | -0.008 | 0.023 |
|  | MPO | -0.122 | -0.085 | -0.048 | -0.046 | -0.013 | 0.019 |
|  | NEO | -0.052 | -0.010 | 0.033 | -0.035 | -0.002 | 0.030 |
|  | AAT | -0.111 | -0.062 | -0.014 | -0.093 | -0.055 | -0.017 |
| start WAZ | Intercept | -0.840 | -0.390 | 0.121 | -1.082 | -0.632 | -0.115 |

**Pathogen nodes**

| **RESPONSE** | **PREDICTOR** | **Age 1**  **95% credibility interval** | | | **Age 2**  **95% credibility interval** | | |
| --- | --- | --- | --- | --- | --- | --- | --- |
|  |  | **2.5%** | **50%** | **97.5%** | **2.5%** | **50%** | **97.5%** |
| Pathogen1 | Intercept | -4.293 | -4.124 | -3.965 | -3.847 | -3.677 | -3.518 |
| Pathogen2 | Intercept | -2.901 | -2.630 | -2.379 | -2.496 | -2.232 | -1.980 |
| Pathogen3 | Intercept | -4.127 | -3.751 | -3.372 | -3.484 | -3.112 | -2.729 |
| Pathogen4 | Intercept | -4.472 | -3.933 | -3.438 | -3.805 | -3.276 | -2.749 |
| Pathogen5 | Intercept | -4.439 | -3.930 | -3.402 | -2.233 | -1.756 | -1.262 |

**AGP and LMZ nodes**

| **RESPONSE** | **PREDICTOR** | **Age 1**  **95% credibility interval** | | | **Age 2**  **95% credibility interval** | | |
| --- | --- | --- | --- | --- | --- | --- | --- |
|  |  | **2.5%** | **50%** | **97.5%** | **2.5%** | **50%** | **97.5%** |
| AGP | Intercept | 4.211 | 4.510 | 4.825 | 4.381 | 4.616 | 4.868 |
|  | Pathogen1 | -0.049 | -0.004 | 0.044 | -0.027 | 0.026 | 0.081 |
|  | Pathogen2 | -0.010 | 0.002 | 0.015 | -0.020 | -0.003 | 0.015 |
|  | Pathogen3 | -0.024 | 0.027 | 0.077 | -0.014 | 0.038 | 0.092 |
|  | Pathogen4 | -0.113 | -0.045 | 0.020 | 0.011 | 0.083 | 0.155 |
|  | Pathogen5 | -0.044 | 0.010 | 0.066 | -0.042 | -0.010 | 0.022 |
|  | MPO | -0.012 | 0.010 | 0.033 | 0.002 | 0.022 | 0.042 |
|  | NEO | -0.032 | -0.005 | 0.022 | -0.047 | -0.029 | -0.009 |
|  | AAT | -0.034 | -0.005 | 0.026 | -0.022 | 0.000 | 0.024 |
|  | LMZ | -0.006 | 0.017 | 0.040 | -0.021 | -0.002 | 0.017 |
|  | ALRI | 0.035 | 0.185 | 0.345 | -0.200 | -0.046 | 0.105 |
|  | Fever | 0.147 | 0.195 | 0.242 | 0.179 | 0.228 | 0.280 |
| LMZ | Intercept | 0.477 | 1.295 | 2.107 | -0.473 | 0.157 | 0.817 |
|  | Pathogen1 | -0.068 | 0.059 | 0.188 | -0.064 | 0.087 | 0.238 |
|  | Pathogen2 | -0.053 | -0.021 | 0.013 | -0.076 | -0.028 | 0.021 |
|  | Pathogen3 | -0.014 | 0.128 | 0.271 | -0.177 | -0.031 | 0.127 |
|  | Pathogen4 | -0.194 | -0.003 | 0.192 | -0.224 | -0.011 | 0.210 |
|  | Pathogen5 | -0.123 | 0.027 | 0.189 | 0.075 | 0.165 | 0.258 |
|  | MPO | -0.164 | -0.103 | -0.038 | -0.043 | 0.010 | 0.066 |
|  | NEO | -0.070 | 0.004 | 0.077 | -0.028 | 0.025 | 0.081 |
|  | AAT | 0.018 | 0.100 | 0.186 | -0.009 | 0.056 | 0.121 |

Faecal biomarker nodes

| **RESPONSE** | **PREDICTOR** | **Age 1**  **95% credibility interval** | | | **Age 2**  **95% credibility interval** | | |
| --- | --- | --- | --- | --- | --- | --- | --- |
|  |  | **2.5%** | **50%** | **97.5%** | **2.5%** | **50%** | **97.5%** |
| MPO | Intercept | 8.458 | 8.710 | 8.973 | 7.913 | 8.158 | 8.429 |
|  | Pathogen1 | -0.159 | -0.027 | 0.103 | -0.090 | 0.067 | 0.234 |
|  | Pathogen2 | 0.034 | 0.068 | 0.105 | 0.038 | 0.090 | 0.143 |
|  | Pathogen3 | -0.105 | 0.043 | 0.197 | 0.005 | 0.164 | 0.324 |
|  | Pathogen4 | -0.066 | 0.131 | 0.330 | -0.393 | -0.185 | 0.032 |
|  | Pathogen5 | -0.163 | 0.000 | 0.154 | -0.130 | -0.036 | 0.059 |
| NEO | Intercept | 7.236 | 7.651 | 8.046 | 6.987 | 7.385 | 7.793 |
|  | Pathogen1 | -0.136 | -0.024 | 0.094 | -0.282 | -0.142 | 0.003 |
|  | Pathogen2 | -0.010 | 0.020 | 0.054 | -0.069 | -0.025 | 0.021 |
|  | Pathogen3 | -0.123 | 0.006 | 0.137 | -0.201 | -0.061 | 0.081 |
|  | Pathogen4 | -0.352 | -0.177 | -0.004 | -0.298 | -0.107 | 0.094 |
|  | Pathogen5 | -0.256 | -0.118 | 0.032 | -0.157 | -0.072 | 0.016 |
| AAT | Intercept | -1.158 | -0.938 | -0.714 | -1.506 | -1.287 | -1.052 |
|  | Pathogen1 | -0.095 | 0.013 | 0.120 | -0.073 | 0.051 | 0.179 |
|  | Pathogen2 | -0.015 | 0.013 | 0.041 | -0.031 | 0.008 | 0.050 |
|  | Pathogen3 | -0.266 | -0.155 | -0.042 | -0.149 | -0.029 | 0.091 |
|  | Pathogen4 | -0.221 | -0.066 | 0.104 | -0.064 | 0.105 | 0.282 |
|  | Pathogen5 | -0.077 | 0.045 | 0.179 | -0.004 | 0.072 | 0.149 |

**Other nodes**

| **RESPONSE** | **PREDICTOR** | **Age 1**  **95% credibility interval** | | | **Age 2**  **95% credibility interval** | | |
| --- | --- | --- | --- | --- | --- | --- | --- |
|  |  | **2.5%** | **50%** | **97.5%** | **2.5%** | **50%** | **97.5%** |
| Age | Intercept | -0.070 | 0.015 | 0.103 |  |  |  |
| Stools | Intercept | 1.076 | 1.149 | 1.228 | 0.584 | 0.662 | 0.744 |
| ALRI | Intercept | -5.310 | -4.168 | -3.566 | -5.287 | -4.225 | -3.598 |
| Fever | Intercept | -2.121 | -1.380 | -0.656 | -2.349 | -1.608 | -0.866 |

1 Kosek M, Guerrant RL, Kang G, *et al.* Assessment of Environmental Enteropathy in the MAL-ED Cohort Study: Theoretical and Analytic Framework. *Clinical Infectious Diseases* 2014; **59**: S239–47.

2 Kosek M, Haque R, Lima A, *et al.* Fecal Markers of Intestinal Inflammation and Permeability Associated with the Subsequent Acquisition of Linear Growth Deficits in Infants. *Am J Trop Med Hyg* 2013; **88**: 390–6.

3 Plummer M. JAGS: A program for analysis of Bayesian graphical models using Gibbs sampling. In: Proceedings of the 3rd International Workshop on Distributed Statistical Computing (DSC 2003). March. 2003: 20–2.

4 Gelman A. Inference and monitoring convergence. In: Markov chain Monte Carlo in practice. Springer, 1996: 131–43.
